# Two faces of the same coin: a qualitative study of patients’ and carers’ coexistence with chronic breathlessness associated with chronic obstructive pulmonary disease (COPD)

**DOI:** 10.1186/s12904-020-00572-7

**Published:** 2020-05-06

**Authors:** Diana H. Ferreira, Slavica Kochovska, Aaron Honson, Jane L. Phillips, David C. Currow

**Affiliations:** 1 Discipline, Palliative and Supportive Services, Flinders University, GPO Box 2100, Adelaide, SA 5001 Australia; 2 IMPACCT, Faculty of Health, University of Technology Sydney, Ultimo, New South Wales 2007 Australia

**Keywords:** Chronic breathlessness, Chronic obstructive pulmonary disease, Qualitative research, Patients, Carers

## Abstract

**Background:**

Chronic breathlessness is a recognized clinical syndrome that severely impacts patients and carers, who become increasingly restricted in their daily activities. Often, patients become reliant on their carers, who are required to provide constant support. Although individual experiences of breathlessness have been previously investigated, there are few studies exploring contemporaneous experiences of breathlessness of the patient and their carer. This study aimed to understand the experience of severe chronic breathlessness in people with chronic obstructive pulmonary disease (COPD) from the perspective of the patient and carer unit.

**Methods:**

A qualitative study embedded in a randomised, placebo-controlled effectiveness study (RCT) of regular, low-dose (≤32 mg/day), sustained-release morphine for chronic breathlessness associated with COPD. Recruitment occurred between July 2017 and November 2018 in one respiratory and palliative care services, in South Australia. Participants were community-dwelling patients with COPD and severe breathlessness (modified Medical Research Council scale 3 or 4) and their carers. Separate semi-structured interviews were conducted with patients and carers, recorded and transcribed verbatim. Analysis was informed by grounded theory using a constant comparative approach.

**Results:**

From the 26 patients with a carer recruited for the RCT in South Australia, nine were interviewed in their homes. Six patients were men, median age 77 years. Carers were mostly women, who were their wives (*n* = 6), median age 70. Five themes emerged from the data: (1) shrinking world; (2) mutual adaptation; (3) co-management; (4) emotional coping; and (5) meaning in the face of death.

**Conclusion:**

Chronic breathlessness is a systemic condition that permeates all aspects of the patient’s and carer’s lives. Working as a team, patients and carers manage chronic breathlessness to achieve maximal function and well-being. Patients and carers share many aspects of the experience of breathlessness, but the carer seems particularly susceptible to emotional distress. Future chronic breathlessness interventions should target the patient and the carer, both together and separately to address their common and individual needs.

**Trial registration:**

The main trial is registered (registration no. NCT02720822; posted March 28, 2016).

## Background

Chronic breathlessness is defined as persisting breathlessness despite optimal treatment of the underlying causes(s), resulting in disability [1]. Chronic obstructive pulmonary disease (COPD) is the leading cause of chronic breathlessness worldwide and its incidence is increasing [2]. Chronic breathlessness in COPD is a predictor of mortality [3, 4] and is associated with severe disability [5].

Qualitative studies are critical in understanding the perspectives of people living with chronic conditions and/or advanced symptoms, and their carers. Up to this point, the experience of chronic breathlessness in COPD has been explored separately for patients and carers [6]. It is now recognised that patients’ experiences of chronic breathlessness are complex and influenced by a range of physical, psychological, social and existential factors, [7, 8] creating challenges for its diagnosis and management. Previous research has shown that carers are also severely affected by chronic breathlessness with rates of depression and anxiety equal to that of patients [9, 10]. As breathlessness increases, more carer support is required, making this a potentially lonely and stressful role [11].

Importantly, there are many aspects of patients’ and carers’ lived experience of chronic breathlessness associated with COPD that need to be better understood. While individual experiences have been extensively explored, there are fewer studies exploring how each member of a patient-carer unit experiences the same symptom.

Understanding patients’ and their carers’ individual experiences and responses to the *same circumstances* of breathlessness and *how they coexist* could enable identification of factors that contribute to successful coping and adaptation to the symptom for both parties involved. These insights are important for informing new models of care that better meet the needs of this largely invisible palliative population.

## Methods

### Study aim

To understand the experience of living with, and responding to, severe chronic breathlessness in people with COPD from the perspective of the patient and their carer.

### Design

This qualitative study was embedded in a phase III, effectiveness, randomised, placebo-controlled trial (RCT) evaluating sustained-release morphine for people with severe chronic breathlessness and COPD (BEAMS trial) [12]. This qualitative was undertaken to understand the patients’ and their carers’ experiences of breathlessness before commencing on the trial medication.

### Setting and participants

Effectiveness studies ensure that the samples selected mimic as closely as possible the population of interest [13]. Therefore, the patients who had completed the BEAMS trial [12] in metropolitan South Australia were eligible to participate if they had a carer; COPD and chronic breathlessness; a modified Medical Research Council (mMRC) breathlessness score of 3 or 4 corresponding to “stops for breath after walking about 100 metres or stops after a few minutes walking on the level” and “too breathlessness to leave the house or breathlessness when dressing or undressing”, respectively [14, 15]; were in a sufficient state of health to be interviewed; and willing and able to provide informed consent. Participating patients identified the existence of a primary carer, defined as “the person who knew the participant best, provided the most help at home, and could help understand any changes experienced on trial” [16]. All carers of participating patients were eligible to participate, unless they were unavailable or unwilling to do so.

### Research team

The interviews were conducted by a medical practitioner, who was a full-time doctoral student (D.F.) and had formal training in qualitative research methodologies. A.H. is a university researcher with background in data collection and people-centered research designs. S.K. is a university researcher with a linguistics background and experience in communication in clinical and research settings. J.P. is an experienced qualitative researcher with a nursing and palliative care background. D.C. is an experienced palliative care clinical academic whose research focuses on chronic breathlessness.

### Recruitment

Using convenience sampling, potential participants were identified and approached by telephone, by the BEAMS trial study nurses. If interested, they were then contacted by the interviewer (D.F.) for the first time. Participants chose the interview location that was more convenient to them, which could be either at home or at the hospital.

### Data collection

Data collection occurred from July 2017 to November 2018. All verbally consenting participants were visited at home by a single researcher (D.F.), who conducted the interviews. In the beginning of the session, the researcher introduced herself, explained her background and research interests, stated her personal goals for the research project and explained the study in detail to both patient and carer, concurrently. Participants were encouraged to ask questions and discuss concerns about the study before providing written informed consent.

Demographic information was obtained for all participants. Single, face-to-face, semi-structured interviews were conducted separately with patients and their carers. Carers were specifically asked to leave the room where the interview with the patient was taking place. Carers where then interviewed in a separate room, while patients waited in the room they were initially interviewed in. Individual interviews provide a safe and private space for each individual, which facilitates the expression of concerns and emotions [17, 18]. All interviews used topic guides following Rocker et al. [19], adapted to reflect the framework of “total breathlessness” [8] (Additional file 1 and Additional file 2). Topic areas included daily activities, feelings, relationships, concerns, and hope. The patients included in this study were very frail and the research team was particularly mindful about minimising their participation burden, without compromising the methodological quality of the study. While patients and carers were not involved in the study design or recruitment, research nurses with extensive experience recruiting people with chronic breathlessness contributed to the design of the interview guides to ensure they were appropriate, applicable and relevant for the population targeted. A constant comparative approach was used to help identify new concepts emerging from the data that could be explored in subsequent interviews [20].

Interviews were recorded and transcribed verbatim (D.F.). Interview transcripts were not returned to participants in order to minimise burden for this very frail population [21]. Review of interview transcripts is a common technique employed to verify accuracy and correct potential errors but there is evidence to suggest they add little value and have the potential to create complications in the analysis [21]. In this qualitative study, potential misinterpretations were minimized by having a second researcher (A.H.) listen to each interview recording and check the transcription’s accuracy. Participants were only contacted a second time if researchers who oversaw the transcription had any doubts about what participants had said. The researcher conducting the interviews collected field notes during each interview and also kept a reflexive journal with impressions about each participant-researcher interaction. The reflexive journal was compared with data emerging from the interviews, which was compared with data and used strategically to facilitate the interpretation of the findings. Data were collected until reaching a point of saturation (i.e. no new concepts were emerging from the data), as agreed by researchers (D.F., J.P., D.C.).

### Data analysis

Data analysis was conducted in NVivo (V 11.4.0 for Mac) using a constant comparative approach guided by grounded theory principles [20, 22]. Open coding of the interview transcripts was conducted by two researchers (D.F., A.H.). Codes were then grouped into themes (D.F.); each theme was illustrated with several quotes to confirm coding validity (D.F., J.P.). Concepts emerging from the patients’ and carers’ data were further examined for any major differences between the two viewpoints (D.F., J.P., D.C.). A matrix summarised the findings of patients and their carers.

### Ethical considerations

Ethics approval for the main trial and this qualitative study were obtained (Hunter New England Human Research Ethics Committee (HREC) Reference No. 15/12/16/3.06) and the BEAMS trial registered (registration No. NCT02720822, posted March 28, 2016; URL https://clinicaltrials.gov/ct2/show/NCT02720822). Written informed consent was obtained from all participants.

This study is reported following the COREQ framework [23].

## Findings

**Fig. 1:**
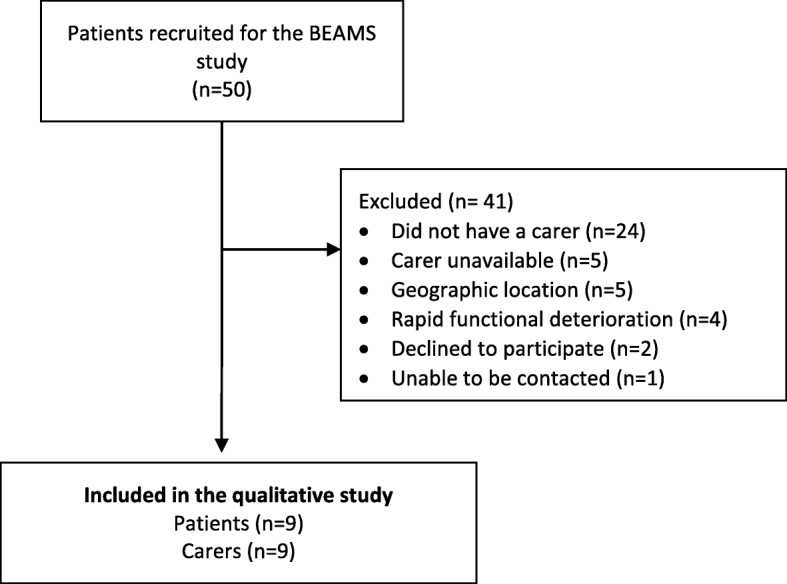
Diagram of patients and carers included

From the 50 patients recruited to the RCT during the study period, only 26 had a carer and were eligible to participate. Of those 26 patients, the carer of five patients could not be interviewed because they were working long hours and a suitable interview time could not be arranged. Of the 21 patients with an available carer, 10 were not included because they were residing more than 70 km away from the city, requiring lengthily travelling for the researchers (*n* = 5); were experiencing rapid functional deterioration and were too frail to be interviewed (*n* = 4); were unable to be contacted (*n* = 1). Of the 11 patients with carers who were approached to participate, two declined participation citing fatigue (Fig. 1).

Nine patients and their carers were separately interviewed in their homes (21–55 min each). Two out of three patients were men and the same proportion of carers were women (and spouses; Table 1). Patients (median age 77 years; interquartile range [IQR] 72–79) were from English-speaking backgrounds and lived in private residences. More than 50% of patients had completed high school. Most patients required occasional or considerable assistance (Table 2).

**Table 1:** Patients (*n* = 9) and their carers (*n* = 9)

Patient & carer ID number	Patient	Carer	Relationship with the patient
1	Female	Male	Husband
2	Male	Female	Wife
3	Female	Male	Son
4	Male	Female	Wife
5	Male	Female	Wife
6	Male	Female	Wife
7	Male	Female	Wife
8	Female	Male	Husband
9	Male	Female	Wife

**Table 2:** Patients characteristics (*n* = 9)

**Median age (IQR)**	77 (72–79)
**Gender**	
Female	3
**Ethnicity**	
Oceanian (Australia or New Zealand)	8
North-West European	1
**Usual language spoken at home**	
English	9
**Residence**	
Living in a private residence	9
**Marital status**	
Married or de facto	8
Widowed	1
**Highest level of education**	
Did not complete high school	4
Completed high school	1
Completed a trade certificate	2
University Degree	2
**mMRC score at baseline**	
3	9
**NRS Worst breathlessness (24 h)**	
4	1
6	2
7	3
8	2
9	1
**AKPS**	
50	3
60	3
70	3

*mMRC* 0–4 modified Medical Research Council scale (higher scores correspond to lower levels of exertion achieved before being limited by breathlessness), *NRS* 0 to 10 numerical rating scale (0 = no breathlessness, *10* worst possible breathlessness); *AKPS* Australia-modified Karnofsky Performance Status (higher scores correspond to better performance status)

Carers (median age 70 years; IQR 69–79) had been living with the patients for many years and were solely providing patients’ care. Seven carers provided hands-on care (median 4 years [IQR 3–5]; Table 3).

**Table 3:** Carer’s characteristics (*n* = 9)

**Median age (IQR)**	70 (69–79)
**Gender**	
Female	6
**Ethnicity**	
Oceanian (Australia or New Zealand)	6
North-West European	3
**Highest level of education**	
Did not complete high school	4
Completed high school	3
University degree	2
**Marital status**	
Married or de facto	9
**Living with the patient**	
Yes	8
No	1
**Median time living with the patient (years) (IQR)**	45 (21–56)
**Hands on care**	
Yes	7
No	2
**For how long has the carer been providing hands-on-care? (median time in years) (IQR)**	4 (3–5)
**Time spent together weekly**	
> 40 h	7
20–40 h	1
10–20 h	1
**Other people involved in providing care**	
No	9
**Changes to employment status due to patient’s condition**	
No	9

The following five themes described the participants’ experience of breathlessness: (1) shrinking world; (2) mutual adaptation; (3) co-management; (4) emotional coping; and (5) meaning in the face of death (Table 4).

**Table 4:** Results of the matrix used for analysis

Patients & Carers	Patient	Carer
1. **Shrinking world** Breathlessness shrinks the physical and relational world available to patients and carers and increases the time they spend together	• Restriction of daily activities • Restriction of independence • Good days and bad days	• Restriction of their own daily activities • Describe patients have good days and bad days • Hard to disconnect from the carer’s role
2. **Mutual adaptation** Patient and carers work individually and together to create optimal functioning for both	• Keep as active as possible • Try to avoid over-exertion • Some push to the limit	• Take over some tasks • Slow down • Step in to prevent perceived over-exertion
3. **Co-management** Patient and carers have active and complementary roles in managing breathlessness	• Have an active role in managing breathlessness • Work with the carer to overcome breathlessness	• Have an active role in managing breathlessness • Degree of participation depends on patients’ limitations • Step in situations of acute breathlessness
4. **Emotional coping** Emotional coping with breathlessness is difficult for patients, but harder for carers	• Annoyance • Frustration	• Frustration • Resentment • Feeling trapped • Giving up part of their identity
5. **Meaning in the face of death** Sense of meaning created by the relationship between patients and carers	• See death as natural (no fear) • Find meaning in their relationship with the carer	• Fear patients’ death and their future • Try to bring joy and happiness to the patient

### Shrinking world

Most patients described that breathlessness significantly impacted their activities of daily living and social lives. Within each patient-carer unit, the level of limitation experienced by carers was highly dependent on the severity of breathlessness experienced by the patients. Carers felt responsible for patients’ wellbeing and spent most of their time with them. Occasionally, carers were forced to leave patients by themselves but they were constantly concerned and found it hard to disconnect from the carer’s role.

*“Well it takes me a long time to get from here to the bed and back. By the time I get there, I got to get on the nebulizer you know? I can get dressed by myself but I have a girl coming to shower me.”* [Patient 1]

*“I have to go shopping and I bought myself a little emergency mobile phone which I haven’t told anybody anything about it. It’s just simply so I can ring home to check on her … And I bought a walker for her hoping that she would use the walker.”* [Carer 1]

As the patient experiences more limitations associated with breathlessness, patient and carer reduce the time they spend apart. In such cases, the carer’s life becomes almost as restricted as the patient. At the same time, there is an attempt to find strategies that enhance patient’s function, allowing the patient-carer unit to perform at a higher level.

*“We went to town about a month ago, we used to do quite a bit of that, become a tourist in your own town, sort of thing. We went there and I walked maybe 50 meters in North Terrace [a street]. I could not go any further. I just could not walk any further. So, that [mobility scooter] will fix our problem. I can even walk to the shops with J. [wife].”* [Patient 9]

*“I would like to go and do things with him, like go to the zoo, the art gallery, places like that but that is out of the question because I couldn’t get the car close enough for him to even get in the place. To be able to walk to get in places. We can’t get around. I don’t go to everything that I would normally go to.”* [Carer 9]

Independently of overall breathlessness severity, both patients and their carers described great daily variability in breathlessness intensity, using terms like “good days” and “bad days” to describe fluctuations and their impact on daily life. Occasionally, they would identify triggers (e.g. temperature, humidity) for such fluctuations, but mostly, they were unpredictable.

“*I have good days and bad days. Some days, I can’t get out of bed basically and other days is fine. ( … ) It is unexpected and you just take it day by day.*” [Patient 8]

*“There are good days and bad days. There are days in which she can’t do much and she just sits down or lays on the bed and does not do much. And other days she has got a lot of energy and does a lot of other stuff. There are quite a few ups and downs. ( … ) In the end you just go with the flow, treat it from day to day, whatever comes up. Yeah.”* [Carer 8]

### Mutual adaptation

Within each patient-carer unit, attempts to reconcile the level of function of the patient with that of the carer were ongoing. While patients tried to be as functional as possible, carers learned how take over certain tasks so as not to allow the patient to over-exert. Both elements worked to keep each patient-carer unit as functional as possible under difficult circumstances.

*“Occasionally, I try to do a bit of cooking. But I can’t always do it. I can’t do any cooking really when I am using this [points at the oxygen tube] because of the fire risk.”* [Patient 6]

*“He can’t do anything which is very annoying - not to me, but to him - because he quite often says … A couple of weeks ago he said “I’ll cook you dinner”. He was going to make a curry. He got up there and I have to switch his oxygen off - because of the gas, you see … Two minutes later he said “I am sorry, I can’t … ” and I said “It’s ok you just sit down and relax and I’ll do it”.* [Carer 6]

In some circumstances, carers learned how to slow down to meet the patient level of function. This strategy seemed to allowed patients to keep as much autonomy as possible.

*“We have a system. T. [son] takes me shopping and I can take a trolley in. And I can do my own shopping like that.”* [Patient 3]

“*Just be patient and let them take their time. When we go to the shops, I don’t rush mum and we just go slow. Be patient.*” [Carer 3]

Within each patient-carer unit, patients and carer had learned to adapt to breathlessness. In most cases, patients had developed an accurate sense of their exertional capacity, learning to push themselves to be as active as possible, while managing their breathlessness, including avoiding situations that created intense or unmanageable breathlessness. In these cases, carers trust the patient capacity to manage the degree of exertion they are able to tolerate.

“*And I think you adapt to the problem. You know you have a problem so you adapt to it. So you create a situation where you don’t get that short of breath, you know? Because you don’t do things.*” [Patient 5]

*“( … ) he sits a lot and plays games on the computer and all that, so he knows when he can’t do things. So yeah, that doesn’t concern me.”* [Carer 5]

In other cases, the carer played an essential role in preventing what was perceived as overexertion causing excess breathlessness.

“*I just don’t want it to beat me. I know my limits and I will push myself to the limits.*” [Patient 7]

*“The last couple of times we used the caravan, it was in the beginning of last year actually. It’s an old caravan so it takes a lot of putting the annex part of it because it doesn’t have a pull-out roof on it and it takes quite a while to put it up, but I have to say “Stop and sit down!” and he says “No, I want to get this done!” and I say “Well, you need to have a rest” so I say “I’ll make you a coffee”. So then he stops. Or we’ll go and have lunch and come back and finish it, you know?”* [Carer 7]

### Co-management

In all patient-carer units, both members had active roles in managing breathlessness. However, carer participation in breathlessness management was largely dependent on the degree of limitations experienced by the patient. In cases where the patient was still reasonably independent, the carer was mainly responsible for more physically-demanding tasks such as shopping or cleaning. In such cases, there was still a clear separation of roles:

*“My wife knows I have a problem and we just get on with it. I do most of the cooking and she does most of the cleaning.”* [Patient 7]

*“I do a few jobs around the house but I don’t have to do anything for him, really. He can dress himself and all things like that. I used to have to cut his toe nails … But now, because we have a care plan, we go to the podiatrist.”* [Carer 7]

In cases where the patient required considerable assistance, carers had a particularly active role. Both patients and carers had developed strategies which allowed them to work as cohesive team to overcome the limitations imposed by breathlessness.

*“Getting dressed is … hands above the head is a problem. But the rest of it is fine, it is just slow. So J. [wife] comes in and helps me and we get through it together.”* [Patient 9]

*“Like we had our granddaughter 21st birthday in the city and it took an hour and a half for him to get dressed. He would do one little thing and then he would get out of breath. And then I would go away and try to put something on myself and come back and do some small thing.”* [Carer 9]

Carers were particularly important in situations of acute, uncontrolled breathlessness, that could be sudden and unpredictable. In some cases, the patient is able to reach for carer’s help but is unable to take further action.

*“When that flu hit me, I couldn’t breathe at all. It was so bad all of a sudden. M. [wife] had to call the ambulance.”* [Patient 4]

“*When he had that flu, I called the ambulance and they came pronto. I know what to do in those cases because I’ve done it before, but it’s a bit scary.”* [Carer 4]

### Emotional coping

While most patients expressed annoyance at being unable to function fully independently, the sensation of daily breathlessness with which they had lived for so long, did not generate distress because they were familiar with the symptom and how to handle it. Conversely, daily breathlessness was an emotional challenge for carers who not only experienced a constant state of worry, but also felt the need to appear calm to patients.

*“No, not scared [by daily breathlessness]. I tend to be a phlegmatic sort of guy; I have low anxiety levels. And I am used to it.”* [Patient 2]

*“Just watching him trying to get his breath … That is horrible to watch. And there is nothing you can do to help. Just keep him comfortable and be there, whatever he needs … I try not to worry too much; I try to keep calm because I don’t want him to get worse.”* [Carer 2]

Conversely, both patients and carers felt emotionally challenged by episodes of acute breathlessness (e.g. COPD exacerbations), which were described as “a worry”, “scary” and “awful”.

*“But a couple of times, a couple of months ago, I had a real breathlessness attack and I could not breathe. It that was quite scary but I managed it ok, I sort of did what I had to and I was ok.”* [Patient 8]

*“It scares me [when she gets more intense breathlessness]. Very much! Especially when she has an infection or something a bit more serious, that is really scary.”* [Carer 8]

While some patients expressed being annoyed with their condition, their carers experienced more intense feelings, such as frustration and resentment, by being forced to step up to the carer’s role. For some, being a carer made them give up a part of their identity, which created a sensation of being trapped in the carer’s role.

*“I get up in the morning, have breakfast, shower. I don’t shower every day because I get so breathless showering. But when I do shower, then M. [wife] helps me sometimes. And then I sit down and I have my Ipad, I look at all the papers from all over the world and I just keep my brain working thinking about things but physically, I don’t do anything at all. ( … ) sometimes I get bored. Bored you know? I think “what should I do next?””*. [Patient 6]

*“My life is boring! [laughs] I am a very outgoing person, I like the outdoors, and on a day like today you think “oh, let’s get in the car and go!” But I have to think of him first because he can’t walk from here to there before he gets breathlessness so I think “well … just scrap it”, and do nothing you know? So it makes it hard on me because I want to do things but I can’t you know?”* [Carer 6]

### Meaning in the face of death

The relationship between patient and carer brought a sense of meaning to both parties and helped to withstand the limitations imposed by chronic breathlessness. For patients, their perspectives on life and death changed with the experience of chronic breathlessness. Many were aware of the severity of their condition but were not afraid to die. Unlike patients, their carers felt more concerned about the future and did not bring up the topic of death easily.

“*No, not anymore [I don’t worry about the future]. I look at it this way, if something is going to happen, it is going to happen. There is nothing you can do about it.”* [Patient 4]

“*I wonder just how bad he’s going to get … What is going to be like further down the track*.” [Carer 4]

Within a patient-carer unit, both patient and carer were essential to ensure the patient kept living a meaningful life. For patients, an essential part of living a good life was being close to their carers. Carers were not only involved in dealing with the physical burdens of breathlessness but they also felt responsible for bringing meaning and joy to patient’s lives.

*“Life is pretty good, yeah. B. [husband] does everything and we are together all the time. He is a good doctor! A very good doctor [laughing].”* [Patient 1]

“*I buy her some flowers every fortnight and I put them in there so she can sit at the table and look at them. Just to introduce a bit of color and a variety into the family. I am trying to make it fairly compatible being at home (and I think for the last year she has been home now) I think she said she was happy I think, yeah.*” [Carer 1]

## Discussion

This is one of the first studies exploring patients and carers co-existence with chronic breathlessness due to COPD. It highlights important issues for patients and carers individually, but also for the patient and carer as a unit. These findings suggest that patients and carers share the experience of chronic breathlessness, working as a team to overcome the restrictions imposed by this syndrome. Importantly, these findings also suggest that *both the patient and the carer* are active participants in the breathing-thinking-functioning cycles of chronic breathlessness [24].

### Breathing

Both patients and carers reported that COPD associated breathlessness took over their lives. While patients experienced breathlessness on a daily basis and had learnt how to accept and adapt to the symptom, carers were frequently distressed by watching patients trying to catch their breath. Previous evidence suggests that carers of people with COPD feel they lack understanding about the experience of breathlessness and its causes [25]. More importantly, they are seldom involved in therapies aiming to optimise lung function and exercise tolerance [25]. Thus, frequent distress with daily breathlessness may be a consequence of lack of education for carers in this area.

Interestingly, the terms “good / bad days” are used by both patients and carers to describe daily fluctuations in breathlessness, in which a “good day” corresponds to being as functional as possible and a “bad day” to severely restricted daily activities. Fluctuations in breathlessness were previously described in a large prospective study examining breathlessness intensity in people with severe COPD, over a 6-month period, in which breathlessness was measured monthly [26]. The current study suggests that fluctuations in breathlessness are frequent, with substantial daily variations. The concept of “good” and “bad” days has been described before in other qualitative work [5]. However, there is no understanding of the predictors of such fluctuations and the mechanisms behind them. These findings highlight the natural history of chronic breathlessness, and the need to develop specific interventions that can target daily fluctuations in breathlessness. Moreover, the descriptions of “good” and “bad” days are consistent between patients and carers, which indicates they share a common experience. Thus, specific interventions targeting “good” and “bad” days should target both the patient *and* the carer *together*.

### Thinking

Within each patient-carer unit, both patients and carers reported distress due to breathlessness, with important differences between both. While patients’ distress is focused on episodes of acute breathlessness which are sudden and unpredictable, carers live in a constant state of worry, which is further exacerbated with sudden episodes of unpredictable breathlessness. Although patients feel regularly annoyed for not being able to fully function, the daily experience of breathlessness does not seem to cause anxiety because it is predictable and manageable. However, unexpected surges of intense breathlessness (e.g. exacerbations of COPD) cause fear and anxiety because they are surprising and difficult to manage. In such situations, fear may itself contribute in aggravating breathlessness [27]. In general, the experience of breathlessness seems to be particularly distressing for carers, who are unable to disconnect from the carer’s role, live in a permanent state of hypervigilance and force themselves to keep calm in order not to disturb patients. On top of that, carers are required to step up in situations of acute unpredictable breathlessness, whether they feel prepared for it or not. While breathlessness-induced emotional burden has been previously described for patients and carers [28, 29], this study adds to the literature by demonstrating that within the same patient-carer unit, similar experiences of breathlessness trigger different emotional responses in patients and carers. More importantly, situations that are not distressing for patients, may be severely distressing for carers. Such findings are important not only because they highlight carers’ psychological vulnerability, but also because there is a significant association between the psychological well-being of patients and their carers [30]. Thus, it is important to implement psychological interventions that target both the patient *and* the carer. Importantly, patients and carers have specific emotional vulnerabilities that may require specific, *individual* approaches.

It is also important to note that patients’ speech is underpinned by a sense of acceptance and adaptation to their condition. Patients acknowledge they may deteriorate and talk easily about dying. This acceptance may be related with the progressive nature of COPD providing time to process and adapt to new challenges, and the perception of having a self-inflicted disease (i.e. believing they are just living with the consequence of their actions) [31, 32]. On the contrary carers find it difficult to accept their role. They feel forced to give part of their identity, trapped in the carers role and have difficulties discussing death-related themes [11]. Thus, coping to the long-term consequences of breathlessness is also more challenging for carers, which may require specific interventions for carers.

### Functioning

In this study, patients and carers reported that they saw their worlds shrink physically and socially due to chronic breathlessness. This limitation of each person’s “living space” [5] has a profound impact on patients’ and carers’ wellbeing. Patient and carer become confined to activities at home and increasingly isolated [33]. This study highlights the concept of “mutual adaptation” in which there is an attempt to reconciliate the patient and the carer level of function so they can function *together*. As breathlessness progresses, patients become more accepting of their limitations [34], but they constantly strive to be as functionally independent as possible. For people with advanced diseases, not being a burden to their families [35] is often more important than having their symptoms under control. This helps to explain why patients are constantly working to their physical (and psychological) limits. Conversely, carers learn to either take over certain tasks or slow down to adapt to the patients’ rhythm. Although a change in life rhythm has been previously reported in patients with COPD and their partners, that consisted mostly on carer’s compromise to adapt to the patient’s limitations [36]. This study suggests both carer and patient play an active role in this process.

Another important finding is that carers may be key intervenients to prevent over-exertion. It has been described that patients adapt to breathlessness by avoiding situations that may trigger unmanageable breathlessness and by careful planning each daily activity to minimize the consequences of breathlessness [31]. This study supports these findings by showing that patients push to their perceived limit while avoiding a degree of exertion that would trigger uncontrollable breathlessness. However, in some circumstances, there is a mismatch between patients and carers perceptions on what constitutes the “limit”. One hypothesis is that the carer’s perception of breathlessness is inaccurate, which is supported by previous research showing only fair patient-carer agreement in breathlessness ratings [37]. Alternatively, the carer may indeed play a crucial role in preventing over-exertion. The presence of a carer may also contribute to increase patient’s confidence to push themselves because there is help available if breathlessness becomes uncontrollable. These hypotheses need to be investigated in future work. Nevertheless, given that breathlessness is managed by patient and carer *together*, it is important to conciliate patient’s and carer’s views on what constitutes over-exertion.

This study also shows that patient and carer are both actively involved in managing breathlessness. Initially, breathlessness is managed by sharing tasks. At this point there is still a clear definition between the patient and the carer roles. This study shows that as breathlessness progresses, patient and carer transition to managing breathlessness as a cohesive team, which requires flexibility and constant adaptation by both parties. This is in line with previous work describing the lack of definition and unpredictability associated with the carer role in severe COPD [38].

Overall, both patient and carer work (individually and together) to withstand the limitations imposed by breathlessness. Carers, and particularly family carers, are a source of physical and emotional support for patients with advanced COPD [39]. Close relationships are essential in creating meaning in people’s lives, contributing to their psycho-social well-being and, ultimately, their quality of life [40]. Because breathlessness leads to social isolation, the carer is frequently the only person providing a sense of connectedness with another person [41]. Thus, the carer is not only the person providing care but also the person providing meaning, and as such is essential in enhancing optimal co-existing with breathlessness. Given that breathlessness impacts on both patients and carers, and both elements play a role in breathlessness management, it was suggested that the patient-carer unit should be targeted as the unit of care [42]. This work supports this idea. However, it also highlights that patients and carers have specific needs, which need to be addressed separately.

### Limitations

Although patients and carers were interviewed separately, they were aware of each other’s presence in the house, which may have inhibited the expression of concerns. All interviews were conducted by the same interviewer (D.F.) for whom English is not a first language. Potential misinterpretations, however, were minimised by having a second researcher (A.H.), whose first language is English, check all interview transcriptions for accuracy. This is a qualitative study so its findings cannot be generalised. However, data checking and analyses were conducted by different researchers to minimise risk of bias. Furthermore, this study was embedded in a RCT and patients were asked to recall their experiences of breathlessness before the intervention was administered. The possibility of recall bias needs to be considered. Importantly, the main trial was an effectiveness study as opposed to an efficacy study. Effectiveness studies in this setting have demonstrated to have high external validity for the population of interest [13, 43]. This study included a high proportion of patients who had completed secondary education, which may not reflect the totality of the population seen in clinical practice. Patients who were living more than 1 hour away from the city centre were excluded due to lengthy travelling times, which may have excluded the perspectives of people who living in more remote areas. This study does not inform us about people with severe COPD who do not (and potentially could not) identify a carer.

### Implications for future research

Chronic breathlessness was described as the product of breathing, thinking and functioning vicious cycles that generate and aggravate the syndrome [24]. This study identified that both patient and caregiver are active participants in those cycles. Framing patients’ and carers’ interventions in accordance with the breathing-thinking-functioning model of breathlessness facilitates the identification of factors that contribute to aggravate and ameliorate each cycle [24]. The complete identification of such factors may ultimately aid to develop an intervention programme that is appropriate for patient, carer and the patient-carer unit as a whole.

This study also underlines the importance of conducting future dyadic studies in the setting of chronic breathlessness, which not only examine the separate perspectives of patients and carer but also *how their relationship facilitates coping with breathlessness* and *what type of support they require*.

### Implications for clinical practice

This study identifies crucial aspects of the patients’ and their carers’ experience with severe chronic breathlessness associated with COPD. Recommendations for clinical practice include:

- Functional losses due to breathlessness are devastating for patients and carers and should be proactively investigated in all stages of COPD.
- Understanding the natural history of COPD and its impact on daily fluctuations in chronic breathlessness may assist in tailoring future interventions for chronic breathlessness, especially self-management strategies involving patient and carer.
- Carers are severely affected by the experience of chronic breathlessness and their specific needs need to be assessed in clinical practice. From a service point of view, the carer is both a care recipient and a co-worker with health professionals [44].
- Both patient and carer are essential in the effective management of chronic breathlessness. Both need to be targeted (together and separately) to optimise clinical outcomes in breathlessness associated with COPD.

## Conclusions

Patients with COPD associated breathlessness and their carers report that breathlessness permeates all aspects of life. Patient and carer see their world shrink and cooperate to create optimal function for the patient-carer unit. A new dynamic is created with patients trying to be as active as possible, and carers trying to be less active so their partners can keep up. Involving both the patient and their carer is essential to create optimal management of chronic breathlessness. Chronic breathlessness is particularly heavy on carers, who feel constantly anxious and have difficulties adapting to their role. Interventions targeting the patient-carer unit but also the carer individually are suggested.

## Supplementary information

**Additional file 1.**

**Additional file 2.**

## Data Availability

The data of this study are available from the corresponding authors on reasonable request.

## Abbreviations

COREQ: Consolidated criteria for reporting qualitative research
COPD: Chronic Obstructive Pulmonary Disease
RCT: Randomised controlled trial
mMRC: modified Medical Research Council scale
HREC: Human Research Ethics Committee
IQR: Interquartile range
NRS: Numerical Rating Scale
AKPS: Australia-modified Karnofsky Performance Status.

## References

[1] Johnson MJ, Yorke J, Hansen-Flaschen J, Lansing R, Ekström M, Similowski T (2017). Towards an expert consensus to delineate a clinical syndrome of chronic breathlessness. Eur Respir J.

[2] Celli BR, MacNee W, Agusti A, Anzueto A, Berg B, Buist AS (2004). Standards for the diagnosis and treatment of patients with COPD: a summary of the ATS/ERS position paper. Eur Respir J.

[3] Nishimura K, Izumi T, Tsukino M, Oga T (2002). Dyspnea is a better predictor of 5-year survival than airway obstruction in patients with COPD. Chest..

[4] Pesola GR, Ahsan H (2016). Dyspnea as an independent predictor of mortality. Clin Respir J.

[5] Ek K, Sahlberg-Blom E, Andershed B, Ternestedt BM (2011). Struggling to retain living space: patients’ stories about living with advanced chronic obstructive pulmonary disease. J Adv Nurs.

[6] Hutchinson A, Barclay-Klingle N, Galvin K, Johnson MJ (2018). Living with breathlessness: a systematic literature review and qualitative synthesis. Eur Respir J.

[7] Burki NK, Lee LY (2010). Mechanisms of dyspnea. Chest.

[8] Kamal AH, Maguire JM, Wheeler JL, Currow DC, Abernethy AP (2011). Dyspnea review for the palliative care professional: assessment, burdens, and etiologies. J Palliat Med.

[9] Jácome C, Figueiredo D, Gabriel R, Cruz J, Marques A (2014). Predicting anxiety and depression among family carers of people with chronic obstructive pulmonary disease. Int Psychogeriatr.

[10] Bernabeu-Mora R, García-Guillamón G, Montilla-Herrador J, Escolar-Reina P, Garcia-Vidal JA, Medina-Mirapeix F (2016). Rates and predictors of depression status among caregivers of patients with COPD hospitalized for acute exacerbations: a prospective study. Int J Chronic Obstr.

[11] Bergs D (2002). ‘The hidden client’–women caring for husbands with COPD: their experience of quality of life. J Clin Nurs.

[12] Currow D, Watts GJ, Johnson M, McDonald CF, Miners JO, Somogyi AA (2017). A pragmatic, phase III, multisite, double-blind, placebo-controlled, parallel-arm, dose increment randomised trial of regular, low-dose extended-release morphine for chronic breathlessness: breathlessness, exertion and morphine sulfate (BEAMS) study protocol. BMJ Open.

[13] Matsuoka H, Allingham S, Fazekas B, Brown L, Vandersman Z, Clark K (2019). Comparability of the Australian National Cancer Symptom Trials (CST) Group’s study populations to National Referrals to non-CST specialist palliative care services participating in the palliative care outcomes collaboration. J Pain Symptom Manag.

[14] Bestall JC, Paul EA, Garrod R, Garnham R, Jones PW, Wedzicha JA (1999). Usefulness of the Medical Research Council (MRC) dyspnoea scale as a measure of disability in patients with chronic obstructive pulmonary disease. Thorax.

[15] Stenton C (2008). The MRC breathlessness scale. Occup Med (Lond).

[16] Farquhar M (2016). Supporting informal carers. Palliative Care in Respiratory Disease (ERS Monograph).

[17] Swetenham K, Tieman J, Butow P, Currow D (2015). Communication differences when patients and caregivers are seen separately or together. Int J Palliat Nurs.

[18] Laidsaar-Powell RC, Butow PN, Bu S, Charles C, Gafni A, Lam WW (2013). Physician–patient–companion communication and decision-making: a systematic review of triadic medical consultations. Patient Educ Couns.

[19] Rocker G, Young J, Donahue M, Farquhar M, Simpson C (2012). Perspectives of patients, family caregivers and physicians about the use of opioids for refractory dyspnea in advanced chronic obstructive pulmonary disease. CMAJ.

[20] Boeije H (2002). A purposeful approach to the constant comparative method in the analysis of qualitative interviews. Qual Quant.

[21] Hagens V, Dobrow MJ, Chafe R (2009). Interviewee transcript review: assessing the impact on qualitative research. BMC Med Res Methodol.

[22] Glaser BG, Strauss AL, Strutzel E (1968). The discovery of grounded theory: strategies for qualitative research. Nurs Res.

[23] Tong A, Sainsbury P, Craig J (2007). Consolidated criteria for reporting qualitative research (COREQ): a 32-item checklist for interviews and focus groups. Int J Qual Health Care.

[24] Spathis A, Booth S, Moffat C, Hurst R, Ryan R, Chin C (2017). The breathing, thinking, functioning clinical model: a proposal to facilitate evidence-based breathlessness management in chronic respiratory disease. NPJ Prim Care Respir Med.

[25] Farquhar M, Penfold C, Benson J, Lovick R, Mahadeva R, Howson S, et al. Six key topics informal carers of patients with breathlessness in advanced disease want to learn about and why: MRC phase I study to inform an educational intervention. PLoS One. 2017;12(5):e0177081. 10.1371/journal.pone.0177081. 10.1371/journal.pone.0177081 PMC5419601 28475655

[26] Gysels M, Higginson IJ (2010). The experience of breathlessness: the social course of chronic obstructive pulmonary disease. J Pain Symptom Manag.

[27] Lansing RW, Gracely RH, Banzett RB (2009). The multiple dimensions of dyspnea: review and hypotheses. Respir Physiol Neurobiol.

[28] Gysels M, Bausewein C, Higginson IJ (2007). Experiences of breathlessness: a systematic review of the qualitative literature. Palliat Support Care.

[29] Bailey PH (2001). Death stories: acute exacerbations of chronic obstructive pulmonary disease. Qual Health Res.

[30] Mi E, Mi E, Ewing G, Mahadeva R, Gardener AC, Butcher HH (2017). Associations between the psychological health of patients and carers in advanced COPD. Int J Chron Obstruct Pulmon Dis.

[31] Nicholls DA (2003). The experience of chronic breathlessness. Physiother Theory Pract.

[32] Lindqvist G, Hallberg LR (2010). ‘Feelings of guilt due to self-inflicted disease’ a grounded theory of suffering from chronic obstructive pulmonary disease (COPD). J Health Psychol.

[33] Gabriel R, Figueiredo D, Jácome C, Cruz J, Marques A (2014). Day-to-day living with severe chronic obstructive pulmonary disease: towards a family-based approach to the illness impacts. Psychol Health.

[34] Caress A, Luker K, Chalmers K (2010). Promoting the health of people with chronic obstructive pulmonary disease: patients’ and carers’ views. J Clin Nurs.

[35] Heyland DK, Dodek P, Rocker G, Groll D, Gafni A, Pichora D (2006). What matters most in end-of-life care: perceptions of seriously ill patients and their family members. CMAJ.

[36] Ek K, Ternestedt BM, Andershed B, Sahlberg-Blom E (2011). Shifting life rhythms: couples’ stories about living together when one spouse has advanced chronic obstructive pulmonary disease. J Palliat Care.

[37] Mi E, Mi E, Ewing G, White P, Mahadeva R, Gardener AC (2018). Do patients and carers agree on symptom burden in advanced COPD?. Int J Chron Obstruct Pulmon Dis.

[38] Bove DG, Zakrisson AB, Midtgaard J, Lomborg K, Overgaard D (2016). Undefined and unpredictable responsibility: a focus group study of the experiences of informal caregiver spouses of patients with severe COPD. J Clin Nurs.

[39] Gardiner C, Gott M, Payne S, Small N, Barnes S, Halpin D (2010). Exploring the care needs of patients with advanced COPD: an overview of the literature. Respir Med.

[40] Strang S, Strang P (2001). Spiritual thoughts, coping and ‘sense of coherence’ in brain tumor patients and their spouses. Palliat Med.

[41] Lin HR, Bauer-Wu SM (2003). Psycho-spiritual well-being in patients with advanced cancer: an integrative review of the literature. J Adv Nurs.

[42] Farquhar M (2017). Carers and breathlessness. Curr Opin Support Palliat Care.

[43] Patsopoulos NA (2011). A pragmatic view on pragmatic trials. Dialogues Clin Neurosci.

[44] Stajduhar KI, Nickel DD, Martin WL, Funk L (2008). Situated/being situated: client and co-worker roles of family caregivers in hospice palliative care. Soc Sci Med.

