# Supplementary material for: Two faces of the same coin: a qualitative study of patients’ and carers’ coexistence with chronic breathlessness associated with chronic obstructive pulmonary disease (COPD)
Source: BMC Palliat Care. 2020 May 6;19:64. doi: 10.1186/s12904-020-00572-7 (PMC7203967; doi:10.1186/s12904-020-00572-7)
Supplement: Supplementary file 1 — Additional file 1. [file 12904_2020_572_MOESM1_ESM.docx]

# Appendix 1

# BEAMS Study

# Patient’s and Caregiver’s Sub-study

# ***Interview guide***

# **(Patients)**

**Participant Screening Number _ _ / _ _ / _ _ _**  **Date** _ _ / _ _ / _ _ _ _

**Drop Out □ Study Stage _____**

**Conclusion □**

**You were proposed to participate in this study because you have COPD and feel short of breath…**

**Before taking the study medication…**

**Did shortness of breath affect your life? In what way?**

- Were there any activities you could not do?

(e.g.: Get up, get dress, have a shower, cook, clean the house, do the loan, go out and have a walk, drive, sleep)

- From the things you were not able to do, which ones were the most important to you?
- How did you generally feel when you had shortness of breath? (Well adapted, anxious, concerned…)
- Did your relationships change because of your shortness of breath? (ie: Spouse, family members, friends) If so, in what way?
- For you, what was the worst thing about being short of breath?
- What were your hopes for the future?
- Did you have any fears or concerns about the future?
- Overall, do you think your quality of life changed as a result of being short of breath?
